# A Modified Perfusion Method to Improve the Quality of Procured Donor Pancreas in Rats

**DOI:** 10.4021/gr501e

**Published:** 2012-11-20

**Authors:** Fu Tian Du, Hong Feng Lin, Wei Ding

**Affiliations:** a Department of Hepatobiliary Surgery, Weifang People's Hospital, Weifang 261041, China

**Keywords:** Pancreas transplantation, Donor graft, P-selectin, Intercellular adhesion molecule 1

## Abstract

**Background:**

In this animal study, we evaluated a modified pancreas perfusion method to improve the quality of harvested pancreas in rats. In this model, the portal vein was used as the outflow route during the pancreas perfusion.

**Methods:**

Forty-eight male Wistar rats were randomly divided into study group and control group, with 24 rats in each group. In the study group, the portal vein was used as outflow of perfusion. While in the control group, the post-hepatic vein (right artrium) was used as perfusion outflow. UW solution was used as perfusion and preservation solution. Pancreas tissue samples were collected at 6, 10, and 14 hours after perfusion and cold preserved for histology and immunohistochemistry examination, P-selection (PS) and ICAM-1 were determined. Pancreas samples were also examined using electronic microscope for ultra-structures.

**Results:**

Compared with the study group, in the pancreas of control group there were significant pathological impairments and cellular ultra-structural alterations observed by immunohistochemistry and electronic microscope, and these impairments aggravated with time. There were mild histological alterations in the pancreas of study group.

**Conclusions:**

During the donor pancreas perfusion, the early opening of portal vein as the outflow is better than the opening of the post-hepatic vein for the preservation of donor graft pancreas and the reduction of tissue impairments.

## Introduction

Pancreas transplantation is an effective treatment for type I diabetes mellitus and some type II diabetes mellitus with renal failure, the quality of donor pancreas is an important actor for successful transplantation. The pancreas is a low-blood circulation organ, during the perfusion the outflow route should be established earlier enough to prevent the donor pancreas impairment caused by hypertension of splenic vein [1]. Both the portal vein and post-hepatic vein can be used as outflow route, however, the comparison of these two outflow modalities were rarely seen reported. The purpose of this study was to explore the effect of early opening of portal vein as outflow on the preservation of donor pancreas’ structures and functions, in order to find an optimal perfusion modality and enhance the viability of donor pancreas.

## Materials and Methods

### Animals and reagents

Male Wistar rats, weighing 260 - 300g, were purchased from Experimental Animal Center of China Medical University. The UW pancreas perfusion solution, mouse anti-rat CD34, ICAM-1 monoclonal antibody and rabbit anti-mouse Ig-G were purchased from the Jing Mei Company, China. The ABC immunohistochemistry super-sensitive kit was purchased from Boshde Company, Wu Han, China. The DAB staining kit was purchased from Gene Tech Company.

### Animal grouping and perfusion models

Forty-eight rats were divided into two groups, study group (n = 24) and control group (n = 24). The portal vein was used as perfusion outflow in the study group, and the posterior-hepatic vein was used as outflow in the control group. Rats were fasted for 12 hours prior to the experiment, but were allowed access to water. Rats were anesthetised with ether. An abdominal midline incision was made, intestines were retracted upward, the aortic artery and IVC were isolated bluntly, then the intestines were retracted rightwards and stomach was lifted, the left gastric artery was ligated and dissected, the splenic artery and vein were ligated at the spleen hilum. The bile duct and hepatic artery were isolated, ligated and cut at the liver hilum. The right gastroepiploic artery and vein were ligated, so did the right gastric artery and vein. The superior mesenteric vein was ligated and dissected near the pancreas. The celiac artery circulation was blocked above the branches of celiac trunk using a blocking forcep. The celiac artery below the superior mesenteric artery branch was opened and a perfusion tube was cannulated. Then, perfusion was started, with the speed of 100 ml/h, and perfusate was hung at the height of 50 cm.

In the control group, the right artrium was opened for outflow; in the study group, the portal vein at the bifurcation in the liver hilum was opened for outflow of the preservation solution infusion. When the color of pancreas turned whitish after 5 minutes’ perfusion, the Treitz ligment was cut, the pancreas was separated by blunt dissection from the transverse mesocolon, and the colonic artery and vein were ligated and severed. The proximal duodenum was ligated and cut off from the pylorus, and the distal duodenum was severed at the Treitz ligment. The peripheral tissues around the celiac artery trunk and the celiac artery at the superior mesenteric artery branch were dissected, the celiac artery was isolated, all of the the celiac branches including the lumbar arteries were ligated and dissected. The pancreas with spleen and duodenum were harvested and put into UW solution, preserved at 0 - 4 °C.

### Observations and analysis of pancreas

Pancreas tissue samples were taken from both groups at 6, 10, 14 hours post-perfusion, and were embedded in paraffin and cut into slices, H&E stained for histology examination. P-selectin and ICAM-1 were examined using immunohistochemistry. Pancreas samples were also examined using electronic microscope.

### Statistical analysis

Statistical analysis was carried out using SPSS 13.0 software, the P values of equal to or less than 0.05 were considered statistically significant.

## Results

### Pancreas histology examination

**Figure 1:**
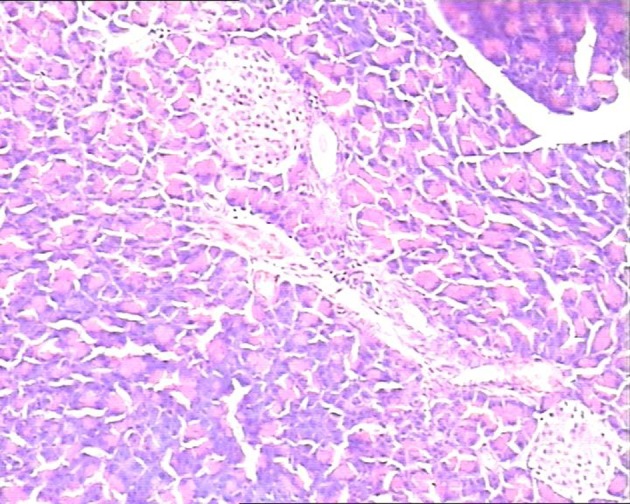
In study group pancreatic acinar structures were normal, and islets were clearly observed located in the center of lobule with normal size. H&E staining, 10 × 10.

**Figure 2:**
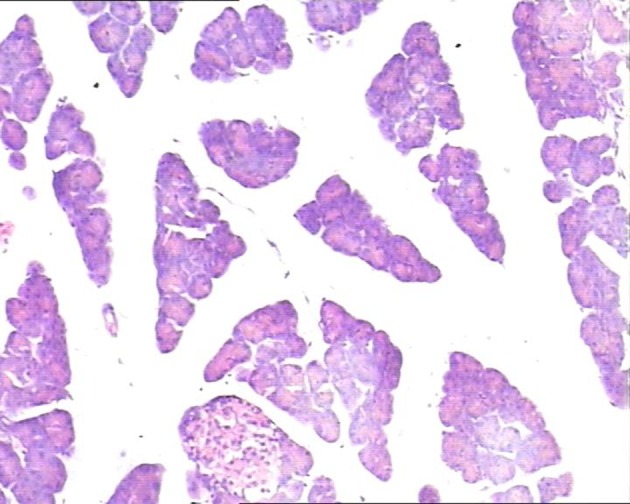
In control group, the pancreatic lobule interstitial space was widening, loose, and edematous. Islets were slightly shrinked with scattered lymphocytes infiltration in the lobules. H&E staining, 10 × 10.

In the control group, there were dilated blood vessels, venous congestion, lobular swelling, and slight shrinkage of islets. The islets septa were loosen and widen. There was lymphocytes infiltration in the interstitial tissue. These histological changes were aggravated with time (Fig. 1). In the study group, the pancreas histology was similar with that of normal pancreas. There was not inflammatory cells infiltration at 6h post perfusion. And, there were slight inflammatory cells infiltration at 10h, and 14h post perfusion. There was no obvious histological impairment (Fig. 2). There was significant difference of the histological changes between the two groups (p < 0.05) (Table 1).

**Table 1:** Histological Examinations (H&E staining)

Time	Group	Venous congestion		Interstitial edema		Inflammation cell infiltration	
		Negative (n)	Positive (n)	Negative (n)	Positive (n)	Negative (n)	Positive (n)
6h* post-perfusion	Study	24	0	24	0	24	0
	Control	19	5	17	7	20	4
10h** post-perfusion	Study	22	2	21	3	23	1
	Control	11	13	10	14	10	14
14h** post-perfusion	Study	21	3	20	4	20	4
	Control	7	17	6	18	3	21

* Compared with control group, Fisher exact probability test, P < 0.05; ** Compared with control group, Chi-square test, P < 0.05.

### Immunohistochemistry

**Figure 3:**
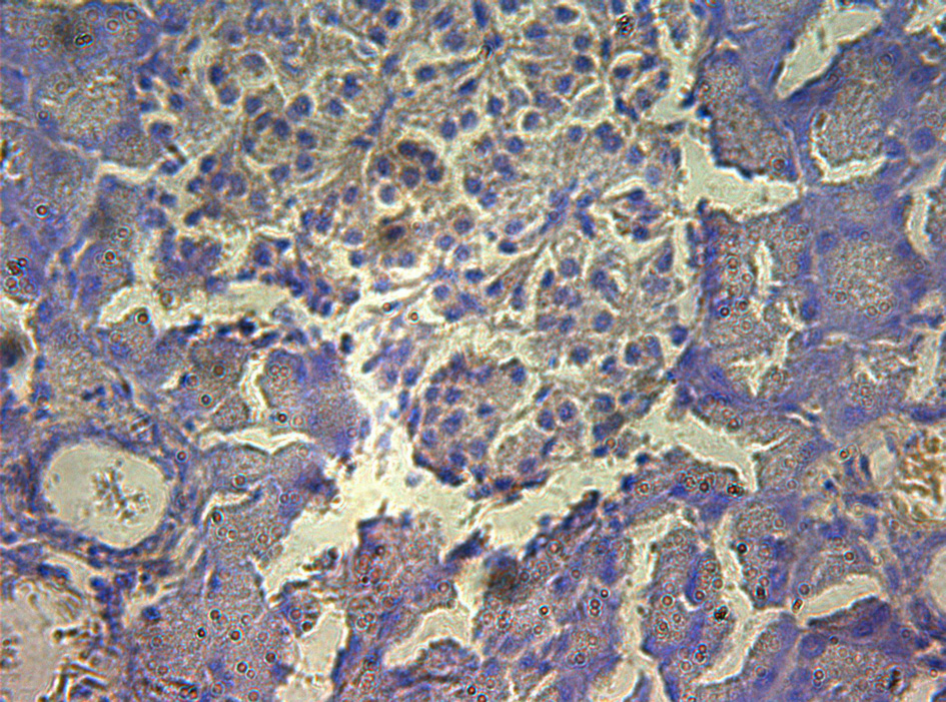
Immunohistochemistry of pancreas in the control group, P-selectin was mildly expressed. 10 × 40.

**Figure 4:**
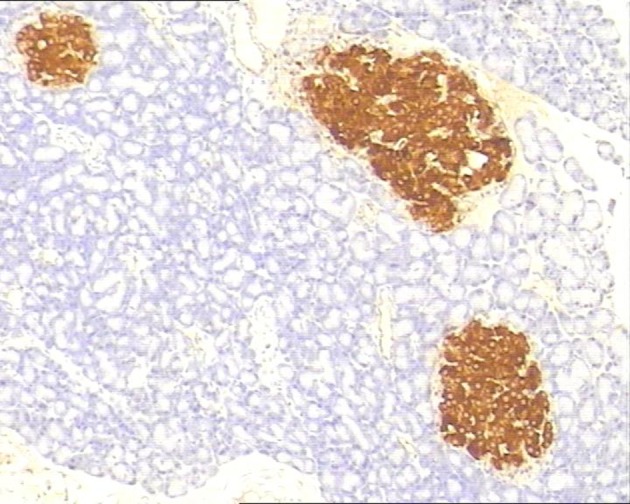
Immunohistochemistry of pancreas in the control group, ICAM-1 was strongly expressed. 10 × 10.

**Figure 5:**
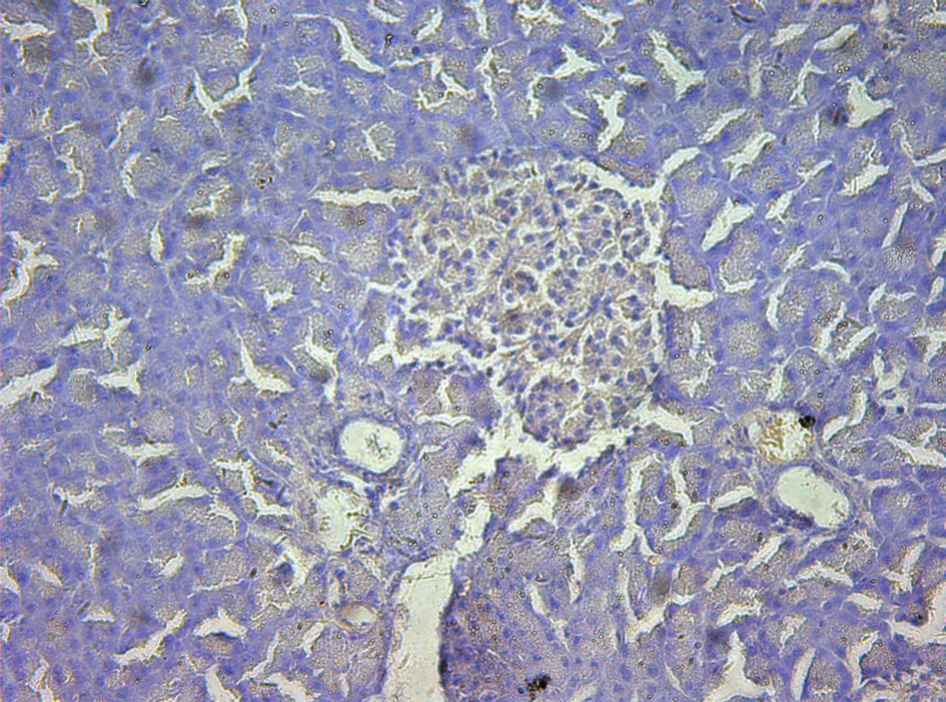
Immunohistochemistry of pancreas in the study group, no P-selectin was expressed. 10 × 20.

**Figure 6:**
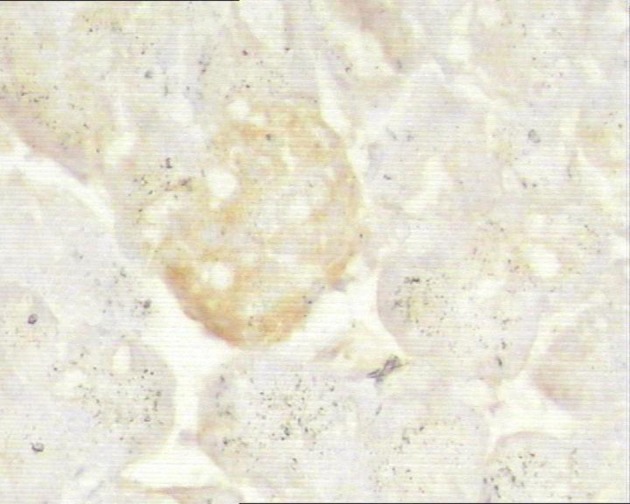
Immunohistochemistry of pancreas in the study group, ICAM-1 was mildly expressed. 10 × 10.

In control group, P-selectin was slightly expressed (Fig. 3). ICAM-1 was expressed in vascular endothelia and islets at most samples after perfusion (Fig. 4). In study group, the P-selectin was not expressed (Fig. 5), some ICAM-1 only slightly expressed with mild positive (Fig. 6), and the others were negative (Tables 2, 3).

**Table 2:** Immunohistochemistry (P-Selectin)

Time	Group	Negative (n)	Mild Positive (n)	Positive (n)
6h* post-perfusion	Study	24	0	0
	Control	20	3	1
10h** post-perfusion	Study	24	0	0
	Control	17	5	2
14h** post-perfusion	Study	22	2	0
	Control	12	8	4

* Compared with control group, Fisher exact probability test, P > 0.05; ** Compared with control group, Chi-square test, P < 0.05.

**Table 3:** Immunohistochemistry (ICAM-1)

Time	Group	Negative (n)	Mild Positive (n)	Positive (n)
6h* post-perfusion	Study	24	0	0
	Control	14	5	5
10h** post-perfusion	Study	23	1	0
	Control	11	6	7
14h** post-perfusion	Study	20	3	1
	Control	7	7	10

* Compared with control group, Fisher exact probability test, P < 0.05; ** Compared with control group, Chi-square test, P < 0.05.

### Electron microscopic study

#### Exocrine cells

In the control group, analysis by electron microscopy, nuclear heterochromatin margination was observed in large amount of exocrine cells, with nuclear chromatin condensation, mitochondrial matrix deeply stained, medullary degeneration, disintegration of cristae. In the study group, there were no obvious changes in most of the exocrine cells. The mitochondrial vacuolar degeneration and medullary degeneration were observed in a small number of cells.

#### B cells

In the control group, most of the islets B cells were with irregular nuclears, and nuclear heterochromatin margination, but normal Golgi apparatus could be found, with mitochondrial medullary degeneration. In the study group, there were no obvious changes in the Golgi apparatus, only with minor mitochondrial medullary degeneration, matrix deeply stained and heterochromatin margination.

## Discussion

Pancreas transplantation can provide insulin-secreting B cells to enhance the insulin secretion, so the recipients can react to the neuro-humoral feedback mechanism, and maintain normal blood glucose level, prevent or delay the development of diabetic complications, and improve quality of life of the diabetic patients [2]. Thus, pancreas transplantation is the most effective treatment for the type I diabetes mellitus and partial type II diabetes mellitus with renal failure [3, 4].

In the pancreas transplantation, the quality of donor pancreas is the key factor for successful transplantation. It is particularly associated with the early stage of post-transplantation complications, such as graft thrombosis, pancreatitis, infection, pancreatic leakage [5]. Pancreas is the second largest secretion organ, differing from the liver and kidneys. Pancreas is a low blood circulation transplantable organ, and very sensitive to tissue enema. During the perfusion, an appropriate outflow should be established promptly to prevent the pancreas impairment caused by high perfusion pressure [6].

Perfusion-related pancreas impairment is the major cause of post-transplantation graft pancreatitis. In recent years, numerous reports showed that the P-selectin and ICAM-1 play important roles in the perfusion-related donor pancreas impairment. P-selectin exists in the alpha granules of platelets and in the Weibel-Palade bodies of endothelial cells. When mediation of thrombin, histamine, tumor necrosis factor or oxygen free radicals, the membranes of alpha granules activate the platelets or endothelial cells, Weibel-Palade bodies merge with the cellular membranes instantly. Then, the P-selectins express in the cellular membranes, which mediate the activation of endothelial cells. P-selectin expression is the initial step of inflammation reaction. P-selectin is not expressed or low-expressed in normal condition, but will be expressed in significantly increasing amount following stimulation of inflammation impairment. P-selectin is not expressed in some organs under normal condition, but will be expressed in the pathological conditions. Thus, the P-selectin expression is considered a marker of some organs’ impairment [7]. ICAM-1, also a kind of cytokine known as CD54, it is the key member of adhesion molecules immunoglobulin gene super family, and it can combine with the CD11/CD18 molecules in the surface of neutrophils. ICAM-1 plays key role for the neutrophils to bind closely with vascular endothelial cells and migrate out of vascular endothelium. ICAM-1 is widely distributed in the body tissues. It is not expressed or in low-expressed in normal condition. When stimulated by certain inflammatory cytokines, it will appear in the surface of many types of cells, and enhance the development and progress of inflammation. Thus the ICAM-1 is also considered a marker of some organs’ impairment.

In the present study, the rat pancreas perfusion model was modified. The perfusion impairment in the control and study groups was evaluated by histology, pathology, cellular ultra-structure observations, and immunohistochemistry for P-selectin and ICAM. The results showed significant differences in pancreas impairment between the two groups, indicating mild impairment of the pancreas in the study group.

Through histological examination, in the control group, the pancreas samples showed dilations of blood vessels, venous congestion and lobular interstitial edema, interstitial lymphocytes infiltration. Moreover, the neutrophils infiltrations and tissue impairment aggravated with time, accompanied by obvious ultra-structural changes under electronic microscope. However, in the study group, the tissue impairments were obviously non-significant, and tissue structures were close to normal with slight ultra-structural changes. It was observed that neutrophils infiltration and tissue structural changes were parallel. Namely, with the increase of neutrophils infiltrations, the pancreatic tissue impairment will aggravate, implying that the neutrophils play an important role in mediating the pancreatic perfusion impairment.

In this study, during the donor pancreas perfusion, we employed the opened portal vein as the perfusate outflow route, which reduced the splenic vein pressure and thus alleviated the donor pancreas perfusion impairment. Consequently, incidences of post-transplantation pancreatitis were reduced or prevented, survival rates of graft pancreas increased. Our results provided experimental data for the donor pancreas procurement with reduced perfusion impairment. More studies are warranted to verify if this perfusion modality could be applied in the clinical donor pancreas harvest procedure.
